# A landscape review of the published research output relating to respiratory syncytial virus (RSV) in North & Central America and Europe between 2011-2015

**DOI:** 10.7189/jogh.09.010425

**Published:** 2019-06

**Authors:** Amir Kirolos, Alex Christides, Shiau Xian, Rachel Reeves, Harish Nair, Harry Campbell

**Affiliations:** Centre for Global Health Research, Usher Institute of Population Health Sciences and Informatics, University of Edinburgh, Edinburgh, Scotland, UK; * Authors contributed equally to this manuscript

## Abstract

**Background:**

The high disease burden of respiratory syncytial virus (RSV) infection and renewed focus on developing a vaccine has led to sustained interest in published RSV-related research. The majority of this research comes from Europe and North/Central America and this landscape review aimed to identify and characterize RSV-related research published during 2011-2015 in these geographical areas.

**Methods:**

We conducted a literature review on electronic databases Scopus and Web of Science to identify published studies investigating RSV throughout Europe and North/Central America. We stratified RSV-related publications between 2011-2015 by study type, country, research institution and funding body.

**Results:**

The annual published output of RSV-related research has increased by 29% over the period 2011-2015. Eighty seven percent (13/15) of the most highly cited papers on RSV during this period were from North America. US universities with the highest number of RSV-related publications included Emory (n = 23), Vanderbilt (n = 23), University of Michigan (n = 21) and Ohio State (n = 20). The UK (n = 125), Netherlands (n = 97) and Spain (n = 76) were major European contributors to RSV-related publications. University Medical Centre Utrecht (n = 40) and Imperial College London (n = 28) were the European universities with the largest number of RSV-related publications. The National Institutes of Health provided funding for one quarter of all RSV-related publications. However, few countries in Eastern Europe, Central America and the Caribbean published RSV-related research. Few epidemiological studies focused on adult populations over 18 years old (n = 28, 7%) with only five publications specifically investigating elderly populations over 65.

**Conclusions:**

This review identifies key regions and research institutions which contributed to RSV-related research during 2011-2015 as well as the donor agencies which supported this research. Further research investment is required in a number of countries. More research in the elderly and in high-risk adults is required given the lack of studies pertaining to these populations. Researchers and those commissioning research can use the data from this review to identify productive research institutions and geographical gaps in RSV research.

Respiratory syncytial virus (RSV) is a major global cause of morbidity, particularly in infants and young children [1]. It is one of the leading causes of acute lower respiratory tract infections in children worldwide, causing over 3.4 million severe infections annually [2]. It is also associated with significant mortality in children in low and lower-middle income countries [1,2], and is increasingly acknowledged as an important pathogen in adults with comorbidities and the elderly [3-5]. As a result, the World Health Organization (WHO) Product Development and Vaccine Advisory Committee (PDVAC) has recognized RSV as the most important future new vaccine target [6]. The increased recognition of the high disease burden from RSV has resulted in an increasing volume of RSV-related research over recent decades [7]. An analysis of RSV-related research output between 1990 and 2013 found that the majority of this research came from high-income countries, particularly Europe and North America [7]. However, no previous review has identified and provided summaries of the current output of RSV-related research by study type, research institution and funding body in these areas. This landscape review provides an overview of RSV-related publications throughout Europe and North/Central America between 2011-2015 to inform the planning of future research in this topic area. This study therefore aimed to determine the types of study and geographical spread of RSV-related publications from Europe and North/Central America. This study also aimed to identify productive research institutions and funding bodies, who contributed to RSV research throughout this time.

## METHODS

### Literature search

Systematic literature searches were conducted on Scopus and Web of Science Core Collection, identified as comprehensive medical databases likely to provide an accurate reflection of RSV-related research output. The main search heading used was Respiratory Syncytial Virus (RSV). Literature searches were limited to European, North American, Central American and Caribbean countries as defined by UN region [8]. Searches were limited to articles published between 1 January 2011 and 31 December 2015. Searches for European countries were undertaken in January 2016 and subsequently for North/Central America in May 2016. The full search strategy is detailed in Appendix S1 of **Online Supplementary Document**. Duplicate articles were identified and removed from the final search result using Endnote (Clarivate Analytics, Philadelphia, PA, USA) initially and subsequently via manual selection by reviewers.

### Study selection

Studies were selected according to the following criteria.

Inclusion criteria:

- Original published research or study relating to RSV;
- Published in European, North American, Central American or Caribbean UN region;
- Study population from European, North American, Central American or Caribbean UN region;
- RSV stated in the title or abstract;
- Study published between 1 January 2011 and 31 December 2015.

Exclusion criteria:

- Editorial material/ letter/ conference paper/ meeting abstract/ expert commentary/ review;
- Study or publication relating to non-respiratory disease;
- Study or publication where RSV is mentioned in the abstract but the study does not contain research related to RSV;
- Study or publication relating to bovine RSV;
- Both first and last authors not from European, North American, Central American or Caribbean country or research institute.

### Data extraction

One study reviewer extracted details of publications into standard templates on Microsoft Excel. These included the publication year, country of the first and last author, affiliated institution of the first and last author, funding source and study population. Where first and last authors were from different countries or institutions, these were both noted. Publications were mapped by geographical location using the online program MapChart (url: https://mapchart.net/).

Publications were categorized as either epidemiological, clinical or laboratory studies. Epidemiological publications included research on the incidence, prevalence and burden of disease of RSV, vaccine research and other public health interventions. Clinical publications included research on risk factors, disease presentation, investigation, treatment and complications. Laboratory publications included research on viral genetics, life cycle and pathogenesis of the disease. Decisions on categorization followed the broad areas listed in **Table 1** and were assigned by reviewers. Where publications covered more than one aspect of RSV research across categories, the main focus of the article (as judged by reviewers) was used to assign publications to a single category.

**Table 1:** Categories of respiratory syncytial virus (RSV)-related publications

Category	Subcategory	Areas included
**Epidemiological publications**	Epidemiology	Incidence, prevalence
	Disease burden	Mortality, morbidity, economic cost
	Disease prevention	Vaccination, surveillance
**Clinical publications**	Predictive or risk factors	Risk factors, prognostic factors, disease severity markers
	Clinical presentation	Signs and symptoms of disease, clinical course of disease
	Investigation	Diagnostic methods
	Management	Treatment of disease
	Complication	Associated complications
**Laboratory publications**	Genetic	Genotypes of virus
	Immunology	Pathogenesis
	Virology	Transcription, transmission of virus

## RESULTS

**Figure 1:**
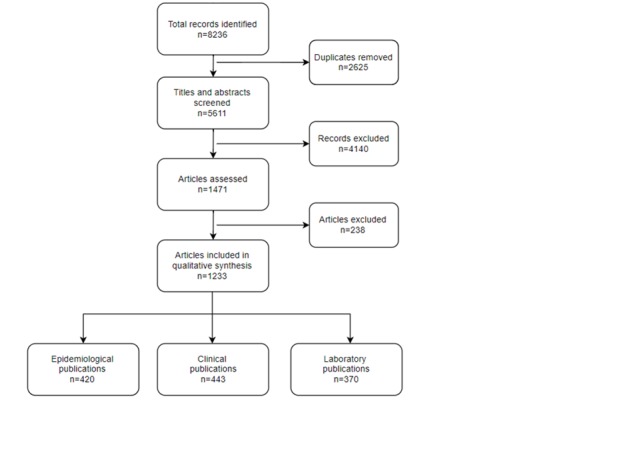
Flowchart of literature review for respiratory syncytial virus (RSV)-related publications.

Publications were identified and screened systematically, as shown in **Figure 1**. After applying the selection criteria, 1233 publications were identified and included in this review. Of publications identified, there were 443 publications relating to clinical studies, 420 publications relating to epidemiological studies and 370 publications relating to laboratory studies.

### Geographical spread of RSV-related publications

**Figure 2:**
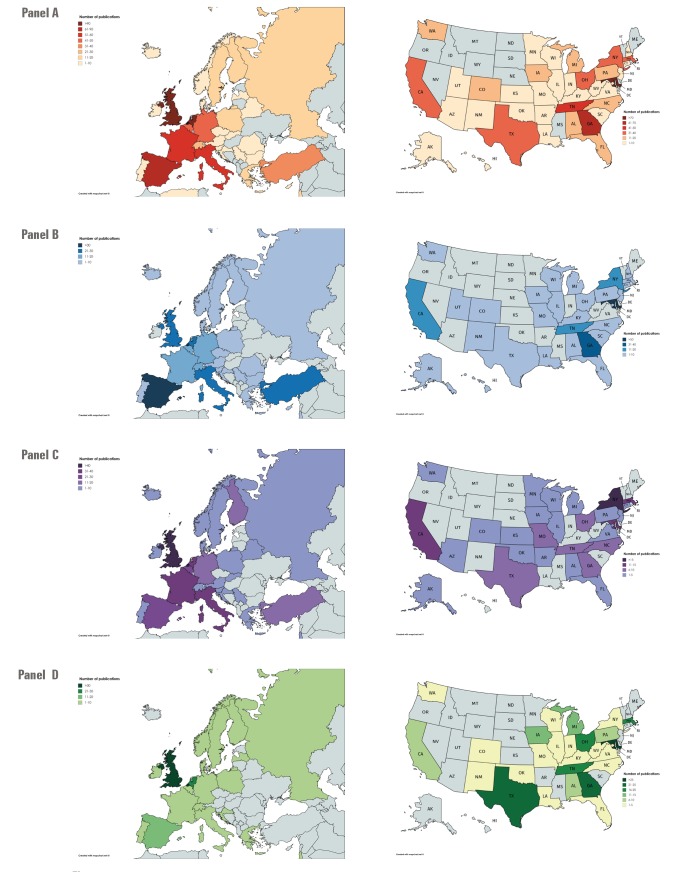
Number of respiratory syncytial virus (RSV) studies published by country in Europe and US state in the USA. **Panel A.** Map showing European countries by number of RSV related publications. **Panel B.** Map showing US states by number of RSV related publications. **Panel C.** Map showing European countries by number of epidemiological RSV publications. **Panel D.** Map showing US states by number of epidemiological RSV publications. **Panel E.** Map showing European countries by number of clinical RSV publications. **Panel F.** Map showing US states by number of clinical RSV publications. **Panel G.** Map showing European countries by number of laboratory RSV publications. **Panel H.** Map showing US states by number of laboratory RSV publications.

**Figure 2** displays maps detailing the number of publications and related study types by country in Europe and by state for the United States of America (USA). In North/Central America, 88% (498/564) of RSV-related publications were from the USA. Maps detailing the number of RSV publications of other countries throughout North America, Central America and the Caribbean are included in Appendix S2 of **Online Supplementary Document**.

The countries which had the most RSV-related publications were USA (n = 498), United Kingdom (UK) (n = 125), Netherlands (n = 97), Spain (n = 76) and France (n = 60) (**Table 2**). The countries with the highest number of RSV-related publications per 100 000 population (according to 2016 World Bank estimates) were Iceland (n = 26.93), Netherlands (n = 5.7), Belgium (n = 4.05), Switzerland (n = 3.34) and Finland (n = 3.09).

**Table 2:** Number of respiratory syncytial virus (RSV) studies published by country and UN region*

Country	Number of RSV publications	Number of RSV publications per 100 000 population (2016 World Bank)		Number of epidemiological RSV publications		Number of clinical RSV publications		Number of laboratory RSV publications
**USA**	498	**1.54**		162		129		207
**UK**	125	**1.90**		28		47		50
**Netherlands**	97	**5.70**		30		40		27
**Spain**	76	**1.64**		37		23		16
**France**	60	**0.90**		16		35		9
**Italy**	58	**0.96**		21		32		5
**Canada**	51	**1.41**		26		14		11
**Belgium**	46	**4.05**		17		22		7
**Germany**	42	**0.51**		17		18		7
**Turkey**	35	**0.44**		22		13		0
**Switzerland**	28	**3.34**		20		5		3
**Sweden**	20	**2.02**		8		9		3
**Finland**	17	**3.09**		2		14		1
**Israel**	16	**1.87**		4		9		3
**Russia**	14	**0.10**		6		7		1
**Poland**	14	**0.37**		6		6		2
**Mexico**	14	**0.11**		7		1		6
**Greece**	11	**1.02**		8		2		1
**Austria**	10	**1.14**		5		5		0
**Denmark**	9	**1.57**		1		7		1
**Iceland**	9	**26.93**		5		4		0
**Croatia**	8	**1.92**		4		1		3
**Norway**	6	**1.15**		2		1		3
**Portugal**	6	**0.58**		2		3		1
**Ireland**	3	**0.63**		0		2		1
**Slovakia**	3	**0.55**		2		1		0
**Bulgaria**	2	**0.28**		2		0		0
**Czech Republic**	2	**0.19**		2		0		0
**Latvia**	2	**1.02**		0		0		2
**Serbia**	2	**0.28**		1		1		0
**Guatemala**	2	**0.12**		2		0		0
**Puerto Rico**	2	**0.59**		2		0		0
**Belarus**	1	**0.11**		0		1		0
**Cyprus**	1	**0.85**		1		0		0
**Romania**	1	**0.05**		1		0		0
**Slovenia**	1	**0.48**		0		1		0
**Trinidad & Tobago**	1	**0.73**		1		0		0
	**RSV publications**		**Epidemiological publications**		**Clinical publications**		**Laboratory publications**	
**Europe**	669 (54%)		224 (53%)		299 (67%)		146 (39%)	
**North/Central America**	564 (46%)		196 (47%)		144 (33%)		224 (61%)	
**Total**	**1233**		**420**		**443**		**370**	

*50 epidemiological studies and 10 clinical studies had first and last authors who were affiliated with different countries. Therefore the total clinical and epidemiological studies in this table add up to a larger number than the total 420 epidemiological and 443 clinical publications reported.

The countries with the most epidemiological publications were the USA (n = 162), Spain (n = 37) and the Netherlands (n = 30). The countries with the most clinical publications were the USA (n = 129), the UK (n = 47) and the Netherlands (n = 40). The countries where the highest number of laboratory studies were published were the USA (n = 207), the UK (n = 50) and the Netherlands (n = 27) (**Table 2**). Over half of RSV-related publications from North/Central America came from five US states; Maryland (n = 94), Georgia (n = 66), Tennessee (n = 41), Texas (n = 39) and Massachusetts (n = 37). Maryland had the largest number of epidemiological publications (n = 52) and laboratory publications (n = 27). New York (n = 19) had the largest number of clinical publications. Appendix S3 of **Online Supplementary Document** contains complete tables of number of RSV related publications by US state.

Europe had 54% (669/1233) of publications compared to North/Central America. Europe produced more epidemiological (53%, 224/420) and clinical publications (67%, 299/443) in comparison to North/Central America; however North/Central America had more laboratory publications (61%, 224/370).

### RSV-related publications by research institution

**Figure 3:**
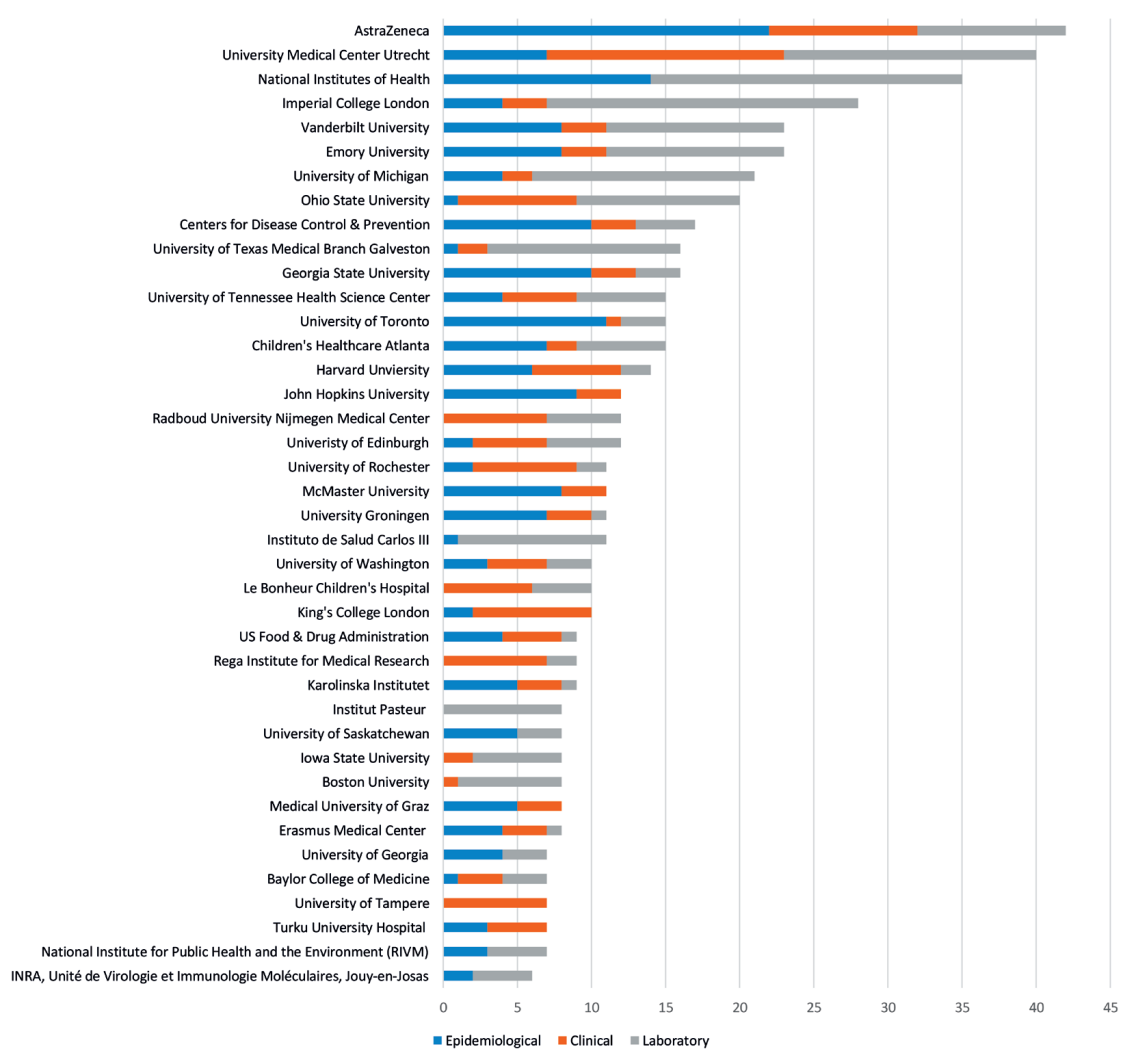
Top institutions that published respiratory syncytial virus (RSV) studies 2011-2015 (number of publications on the horizontal axis). Figure displays only institutes which were identified as having more than five publications.

The number of RSV-related publications from different research institutions and the type of study they published is displayed in **Figure 3** with full tables of results in Appendix S4 of **Online Supplementary Document**. The academic/ not-for-profit institutions that published the most RSV-related articles were University Medical Centre Utrecht (n = 40) and the National Institutes of Health, USA (n = 35). In the commercial/for-profit sector, the institution with the highest number of RSV-related publications was AstraZeneca/MedImmune (n = 42). Overall, AstraZeneca/Medimmune had the most epidemiological RSV publications (n = 22), the University Medical Centre Utrecht had the most clinical RSV publications (n = 16) and the National Institutes of Health had the most laboratory RSV publications (n = 21).

### RSV-related publications by funding body

**Figure 4:**
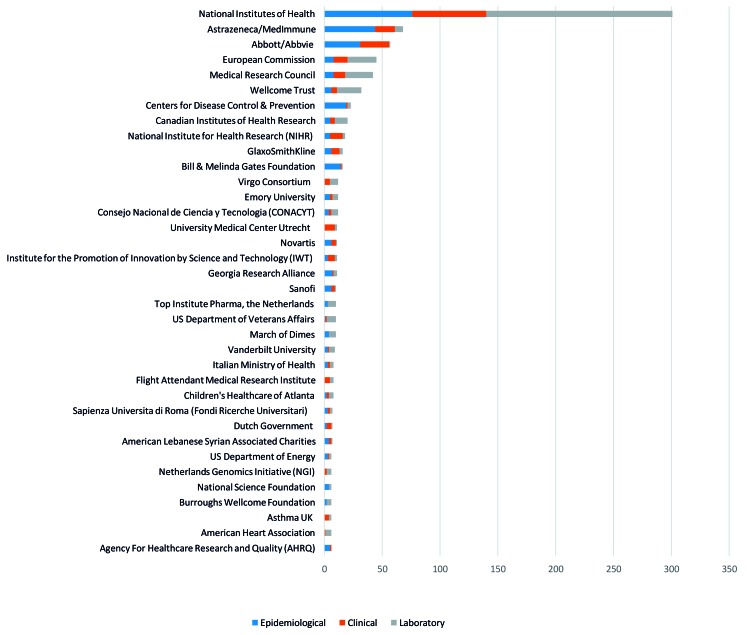
Top agencies providing funding for respiratory syncytial virus (RSV) research by number of RSV studies published 2011-2015. Figure displays only funders which were identified as having funded more than five publications

The funding bodies who provided funding for RSV-related publications in Europe and North/Central America are displayed in **Figure 4** with full tables of results in Appendix S5 of **Online Supplementary Document**. The National Institutes of Health was the agency that provided funding for the largest number of publications (n = 305), as well as within each category (76 epidemiological, 64 clinical and 161 laboratory studies). This is in comparison to other government agencies within Europe such as the European Commission (n = 45) and the UK National Institute for Health Research (UK NIHR) (n = 18). AstraZeneca/Medimmune provided funding for the second highest number of RSV-related publications (n = 68), and funded the second highest number of epidemiological studies (n = 44).

### Epidemiological publications by study population

The majority of epidemiological studies focused on paediatric populations aged <18 years (n = 231, 55%) of which 67 publications focused solely on children aged <1 year. Few epidemiological publications focused on adult populations >18 years (n = 28, 7%). Specifically, of these publications studying adult populations, only five publications studied populations aged over 65. The remaining publications covered either both adult and paediatric populations (n = 46, 11%), were animal studies (n = 84, 20%) or did not specify the focus population of the study (n = 31, 7%).

### RSV-related publications by year in Europe and North/Central America

**Figure 5:**
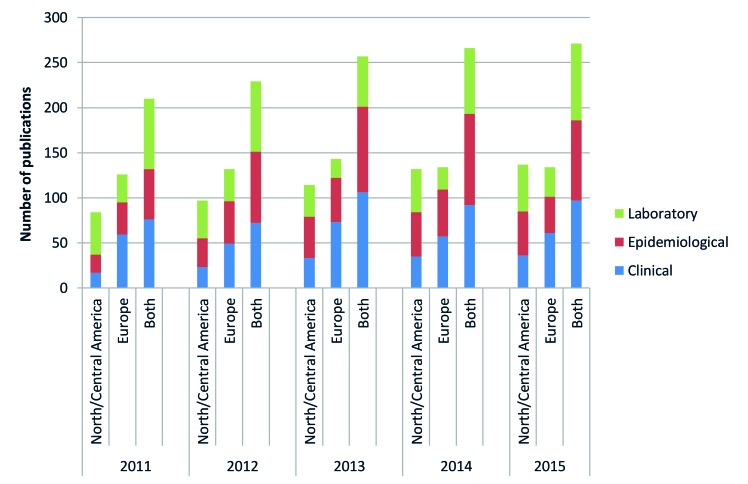
Respiratory syncytial virus (RSV) publications by year, study type and UN region.

**Figure 5** displays the number of RSV-related publications from Europe and North/Central America each year during 2011-2015. The annual number of RSV-related publications increased between 2011 and 2015 by 29% from 210 to 271. Europe produced more RSV-related publications, mostly clinical and epidemiological publications, compared to North/Central America during 2011-2013. However, North/Central America produced the same number of RSV-related publications in 2014 and 2015 and also consistently had more laboratory publications in comparison to Europe.

### Highly cited RSV publications

Fifteen publications published during 2011-2015 with over 100 citations on Web of Science on 12 March 2018 were identified (Appendix S6 of **Online Supplementary Document**). Twelve of these publications were from the USA (one being a collaboration with Spain), with two from the Netherlands and one from Canada.

## DISCUSSION

This landscape review provides an overview of 1233 RSV-related publications from Europe and North/Central America during 2011-2015. This review found that the annual number of RSV-related publications increased by 29% (n = 61) over the period 2011-2015. The USA was the country with the highest number of RSV-related publications (n = 498) with over half of publications from North/Central America coming from five US states (Maryland, Georgia, Tennessee, Texas and Massachusetts). Key US universities publishing the highest number of RSV-related publications included Emory (n = 23), Vanderbilt (n = 23), University of Michigan (n = 21) and Ohio State (n = 20). The UK, Netherlands, Spain and key research institutions, including University Medical Centre Utrecht (n = 40) and Imperial College London (n = 28), were amongst other major contributors to RSV research within this time period. Other countries such as Iceland and Belgium produced a relatively high number of publications in relation to their population size. The National Institutes of Health provided funding for the largest number of RSV-related publications; one quarter of all publications identified in this review. Few or no publications came from a number of countries located throughout Eastern Europe, Central America and the Caribbean. In addition, few epidemiological studies focus on other at risk groups out with infants and children such as the elderly.

The great majority of research was conducted in Western European countries, the USA and Canada. The USA was the country with the largest number of RSV-related publications in each category (clinical n = 129, epidemiological n = 162 and laboratory n = 207) and was associated with the majority of highly cited RSV-related publications (12/15). Within Europe, the UK was the most productive contributor with 125 publications. These major contributions to RSV research from the UK and USA in recent years continue a trend since 1990 where the UK and USA both dominated in terms of RSV-related research output [7].

Consistent with previous work, this review found a significant lack of research in low- and lower-middle income countries [7]. Seven low and lower-middle income countries (Guatemala, Honduras, Nicaragua, Haiti, El Salvador, Georgia and Belize according to 2011 World Bank classifications) were included in this search, and only one of these, Guatemala, published any RSV research (two RSV-related publications) within the study period [9]. No RSV-related research was identified from 43 countries/territories included in the search. There was a noticeable lack of research in several countries in Central America, the Caribbean and Eastern Europe. Greater funding should be invested and academic support should be promoted for clinical and epidemiological RSV research in these countries.

Despite increased vulnerability to RSV infection in the elderly, there was a lack of epidemiological studies which specifically studied elderly populations. The preponderance of studies in infants and children is understandable, given the large burden of disease of RSV in this population and because the majority of vaccine research is focusing on maternal or neonatal vaccination [2]. However, the lack of studies in populations such as the elderly or high-risk adults is surprising given the associated burden of disease and the potential for these populations to benefit from implementation of an RSV vaccine [10].

The National Institutes of Health provided funding for nearly a quarter of all the RSV related publications (n = 301) identified in this review. This is in comparison to other governmental bodies such as the European Commission (n = 45) and UK NIHR (n = 18) and the funder of the second highest number of RSV-related publications - MedImmune/Astrazeneca (n = 68). Overall, governmental agencies such as the National Institutes of Health and the US Centres for Disease Control and Prevention featured prominently as funding bodies along with supranational governmental agencies such as the European Commission. This may be a contributing factor for the aforementioned lack of research output in some countries – with many of the governmental agencies providing funding based in the USA or UK. Pharmaceutical companies also funded a considerable number of studies. Medimmune, whose parent company is AstraZeneca has highly invested in developing an RSV vaccine [11], along with Abbott/Abbvie and Novartis, who were all prominent funders of RSV-related publications.

This study has strengths and limitations. Literature searches were performed systematically in this landscape review on two databases, Web of Science and Scopus. However, it is possible that there are other published studies for RSV-related research that were not included in the databases we searched. It is likely however that given the comprehensive nature of Web of Science and Scopus, these accurately reflect the current makeup of RSV research. This review also only included research from North America, Central American, the Caribbean and Europe. Although it is likely that most of the research will have been undertaken in these locations, this will have missed RSV related research outputs from Asia, Africa, Oceania and South America. The missed continents may have included important research centres in places such as Hong Kong, as well as most of the developing world where RSV is most abundant and has highest morbidity and mortality [2]. Given the high volume of research obtained by this review, quality assessment was not undertaken for the studies identified. Therefore this review characterizes the volume of publications from these areas without commenting on the quality of output. A single study may also result in several publications. Therefore there are assumptions in this review that volume of publications is an appropriate proxy for research output. However, given these limitations over- or under- estimation of research output is a possibility. Analysis of funders was limited to number of studies funded by different bodies and did not account for the size of budgets granted. Publications were categorized by study type using the criteria in **Table 1** by individual reviewers. Therefore these may have been subject to some misclassification bias’. Finally, papers were categorised into countries or research institutes based on the first and last authors as a representation of the main contributors to the publication. While a small number of publications had first and last authors from different countries or institutions, the exclusion of other authors from the author list may have missed evidence of collaborations between other institutions across Europe and North/Central America.

This landscape review provides an overview of RSV research in North/Central America and Europe between 2011-2015, identifying the most productive contributing countries, institutions and funders of RSV research. This review has revealed areas which require further investment, such as epidemiological and clinical studies in Eastern Europe, Central America and the Caribbean. Furthermore, patient populations including high-risk adult and elderly populations may benefit from an increase in RSV-related research. The information from this review will be highly beneficial to those planning and commissioning further RSV research, as well as for those currently involved in RSV-related research who wish to identify research output from other centres.

## Additional material

Online Supplementary Document
