# Supplementary material for: A landscape review of the published research output relating to respiratory syncytial virus (RSV) in North & Central America and Europe between 2011-2015
Source: J Glob Health. 2019 Apr 10;9(1):010425. doi: 10.7189/jogh.09.010425 (PMC6513410; doi:10.7189/jogh.09.010425)
Supplement: Online Supplementary Document [file jogh-09-010425-s001.pdf]

J Glob Health 2019; 9: 010425

A landscape review of the published research output relating to respiratory syncytial virus (RSV) in North & Central America and Europe between 2011-2015 – Appendices

Appendix 1 – Literature review search strategies

North/Central America Scopus search strategy:
(TITLE-ABS-KEY(Respiratory syncytial virus) OR TITLE-ABS- KEY(Respiratory syncytial virus infection) OR
TITLE-ABS- KEY(RSV) OR TITLE-ABS- KEY(RSV infection)) AND PUBYEAR > 2010 AND (LIMIT-
TO(AFFILCOUNTRY,"UnitedStates") OR LIMIT-
TO(AFFILCOUNTRY,"Canada") OR LIMIT-
TO(AFFILCOUNTRY,"Mexico") OR LIMIT-
TO(AFFILCOUNTRY,"Guatemala") OR LIMIT-
TO(AFFILCOUNTRY,"Honduras") OR LIMIT-
TO(AFFILCOUNTRY,"Nicaragua") OR LIMIT-
TO(AFFILCOUNTRY,"Panama") OR LIMIT-
TO(AFFILCOUNTRY,"Haiti") OR LIMIT-TO(AFFILCOUNTRY,"El Salvador") OR LIMIT-TO(AFFILCOUNTRY,"CostaRica") OR LIMIT-
TO(AFFILCOUNTRY,"Trinidad and
Tobago") OR LIMIT-TO(AFFILCOUNTRY,"Cuba") OR LIMIT-
TO(AFFILCOUNTRY,"Greenland") OR LIMIT-
TO(AFFILCOUNTRY,"Belize") OR LIMIT-
TO(AFFILCOUNTRY,"Barbados") OR LIMIT-
TO(AFFILCOUNTRY,"Anguilla") OR LIMIT-
TO(AFFILCOUNTRY,"Antigua and Barbuda") OR LIMIT-
TO(AFFILCOUNTRY,"Aruba") OR LIMIT-
TO(AFFILCOUNTRY,"Bahamas") OR LIMIT-
TO(AFFILCOUNTRY,"Bonaire, Sint Eustatius and Saba") OR LIMIT-
TO(AFFILCOUNTRY,"British Virgin
Islands") OR LIMIT-TO(AFFILCOUNTRY,"CaymanIslands") OR LIMIT-
TO(AFFILCOUNTRY,"Curaçao") OR
LIMIT-TO(AFFILCOUNTRY,"Dominica") OR LIMIT-
TO(AFFILCOUNTRY,"DominicanRepublic") OR LIMIT-
TO(AFFILCOUNTRY,"Grenada") OR LIMIT-
TO(AFFILCOUNTRY,"Guadeloupe") OR LIMIT-
TO(AFFILCOUNTRY,"Jamaica" OR LIMIT-
TO(AFFILCOUNTRY,"Martinique") OR LIMIT-
TO(AFFILCOUNTRY,"Montserrat") OR LIMIT-
TO(AFFILCOUNTRY,"PuertoRico") OR LIMIT-
TO(AFFILCOUNTRY,"Saint-Barthélemy") OR LIMIT-
TO(AFFILCOUNTRY,"Saint Kitts and Nevis") OR LIMIT-

TO(AFFILCOUNTRY,"SaintLucia") OR LIMIT-TO(AFFILCOUNTRY,"SaintMartin") OR LIMIT-TO(AFFILCOUNTRY,"Saint Vincent and the Grenadines") OR LIMIT-TO(AFFILCOUNTRY,"SintMaarten"
) OR LIMIT-TO(AFFILCOUNTRY,"Turks and Caicos Islands") OR LIMIT-TO(AFFILCOUNTRY,"United States
Virgin Islands") OR LIMIT-TO(AFFILCOUNTRY,"Bermuda") OR LIMIT-TO(AFFILCOUNTRY,"Saint Pierre and
Miquelon")

North/Central America Web of Science search strategy:
TOPIC:(Respiratory syncytial virus) OR TOPIC: (Respiratory syncytial virus infection) OR TOPIC: (RSV) OR
TOPIC: (RSV infection)
Refined by:COUNTRIES/TERRITORIES: (USA OR CANADA OR MEXICO OR GUATEMALA OR HONDURAS OR
PANAMA OR EL SALVADOR OR COSTA RICA OR NICARAGUA OR GREENLAND OR GRENADA OR
GUADELOUPE OR HAITI OR TRINID TOBAGO)
Timespan: 2011-2015. Indexes: SCI-EXPANDED, SSCI, A&HCI, CPCI-S, CPCI-SSH, BKCI-S, BKCI-SSH, ESCI,
CCR-EXPANDED, IC.

Europe Scopus Search Strategy:
TITLE-ABS-KEY (Respiratory syncytial virus)
OR TITLE-ABS- KEY (Respiratory syncytial virus infection)
OR TITLE-ABS-KEY (Rsv)
OR TITLE-ABS- KEY (Rsv infection)
AND PUBYEAR> 2010
AND (LIMIT-TO (AFFILCOUNTRY , "United Kingdom")
OR LIMIT-TO (AFFILCOUNTRY , "France")
OR LIMIT-TO (AFFILCOUNTRY , "Spain")
OR LIMIT-TO (AFFILCOUNTRY , "Netherlands")
OR LIMIT-TO (AFFILCOUNTRY , "Italy")
OR LIMIT-TO (AFFILCOUNTRY , "Germany")
OR LIMIT-TO (AFFILCOUNTRY , "Belgium")
OR LIMIT-TO (AFFILCOUNTRY , "Turkey")
OR LIMIT-TO (AFFILCOUNTRY , "Finland")
OR LIMIT-TO (AFFILCOUNTRY , "Sweden")
OR LIMIT-TO (AFFILCOUNTRY , "Switzerland")
OR LIMIT-TO (AFFILCOUNTRY , "Poland")
OR LIMIT-TO (AFFILCOUNTRY , "Israel")
OR LIMIT-TO (AFFILCOUNTRY , "Austria")
OR LIMIT-TO (AFFILCOUNTRY , "Greece")
OR LIMIT-TO (AFFILCOUNTRY , "Denmark")
OR LIMIT-TO (AFFILCOUNTRY , "Russian Federation")

OR LIMIT-TO (AFFILCOUNTRY , "Ireland")
OR LIMIT-TO (AFFILCOUNTRY , "Norway")
OR LIMIT-TO (AFFILCOUNTRY , "Croatia")
OR LIMIT-TO (AFFILCOUNTRY , "Portugal")
OR LIMIT-TO (AFFILCOUNTRY , "Slovenia")
OR LIMIT-TO (AFFILCOUNTRY , "Czech Republic")
OR LIMIT-TO (AFFILCOUNTRY , "Bulgaria")
OR LIMIT-TO (AFFILCOUNTRY , "Romania")
OR LIMIT-TO (AFFILCOUNTRY , "Serbia")
OR LIMIT-TO (AFFILCOUNTRY , "Iceland")
OR LIMIT-TO (AFFILCOUNTRY , "Slovakia")
OR LIMIT-TO (AFFILCOUNTRY , "Estonia")
OR LIMIT-TO (AFFILCOUNTRY , "Cyprus")
OR LIMIT-TO (AFFILCOUNTRY , "Hungary")
OR LIMIT-TO (AFFILCOUNTRY , "Lithuania")
OR LIMIT-TO (AFFILCOUNTRY , "Ukraine")
OR LIMIT-TO (AFFILCOUNTRY , "Georgia")
OR LIMIT-TO (AFFILCOUNTRY , "Latvia")
OR LIMIT-TO (AFFILCOUNTRY , "Azerbaijan")
OR LIMIT-TO (AFFILCOUNTRY , "Belarus")
OR LIMIT-TO (AFFILCOUNTRY , "Kazakhstan")
OR LIMIT-TO (AFFILCOUNTRY , "Monaco")

Europe Web of Science search strategy:
TOPIC: (Respiratory syncytial virus) OR
TOPIC: (Respiratory syncytial virus Infection) OR
TOPIC:(RSV) OR
TOPIC: (RSV infection)
Time Span   2011-2015
Refined by   COUNTRIES/TERRITORIES: (RUSSIA OR AUSTRIA OR ENGLAND OR FRANCE OR NORWAY OR NETHERLANDS OR ITALY OR SPAIN OR LATVIA OR CROATIA OR ESTONIA OR GERMANY OR WALES OR NORTH IRELAN
D OR UK OR UNITED KINGDOM OR BELGIUM OR PORTUGAL OR ICELAND OR FINLAND OR SWEDEN OR SLOVENIA OR CZECH REPUBLIC OR SWITZERLAND OR REUNION OR TURKEY OR SCOTLAND OR ISRAEL OR UKRAINE OR POLAND OR BULGARIA OR ROMANIA OR DENMARK OR SLOVAKIA OR GREECE OR HUNGARY OR CYPRUS OR IRELAND) AND COUNTRIES/TERRITORIES: (ENGLAND OR SLOVENIA OR ICELAND OR FRANCE OR CZECH REPUBLIC OR NETHERLANDS OR REUNION OR ITALY OR SPAIN OR GERMANY OR UKRAINE OR UK OR BELGIUM OR FINLAND OR BULGARIA OR CYPRUS OR SWEDEN OR ROMANIA OR SWITZERLAND OR SLOVAKIA OR TURKEY OR SCOTLAND OR HUNGARY OR ISRAEL OR POLAND OR DENMARK OR GREECE OR IRELAND OR LATVIA OR REP OF GEORGIA OR RUSSIA OR AUSTRIA OR ESTONIA OR NORWAY OR CROATIA OR WALES OR BYELARUS OR NORTH IRELAND OR UNITED KINGDOM OR PORTUGAL)

Appendix 2 – Publications by country throughout North America, Central America and the Caribbean

Figure A2.1 Total RSV publications by country in North/Central America

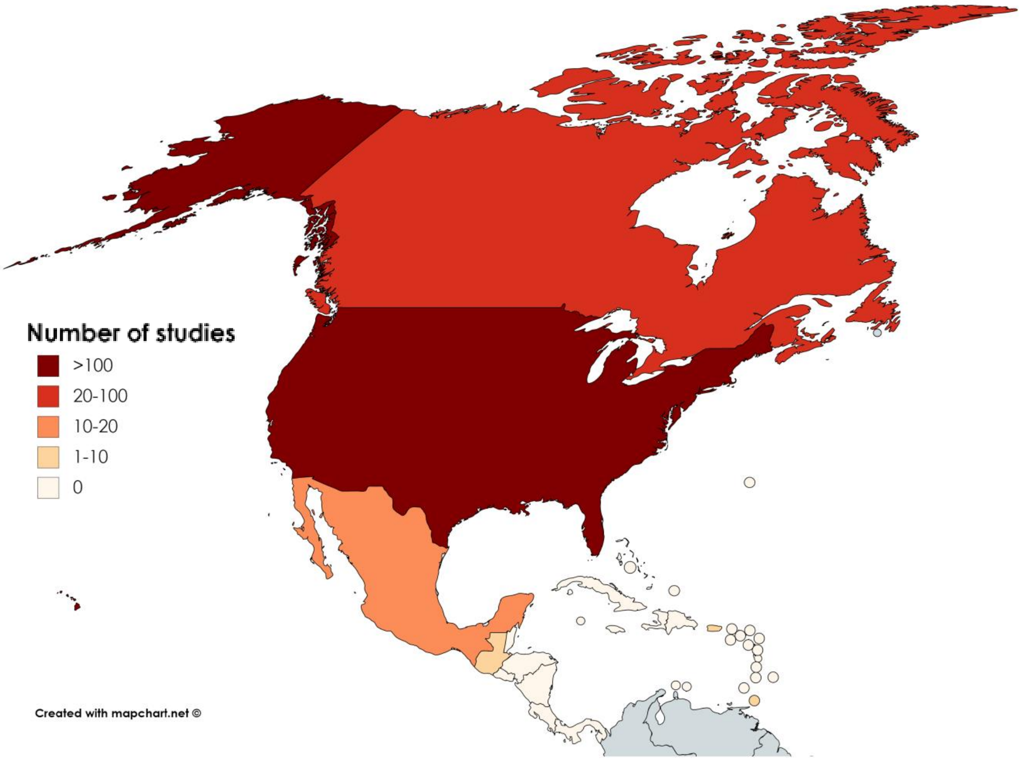

Figure A2.2 Epidemiological RSV publications by country in North/Central America

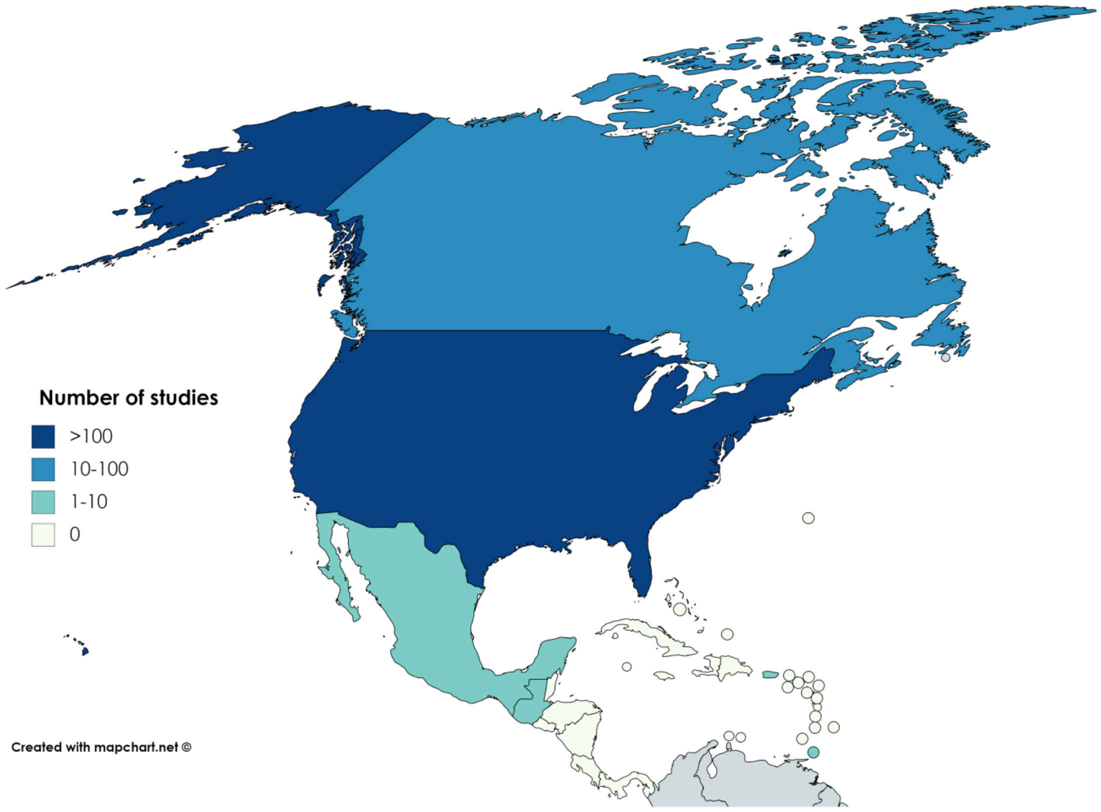

Figure A2.3 Clinical RSV publications by country in North/Central America

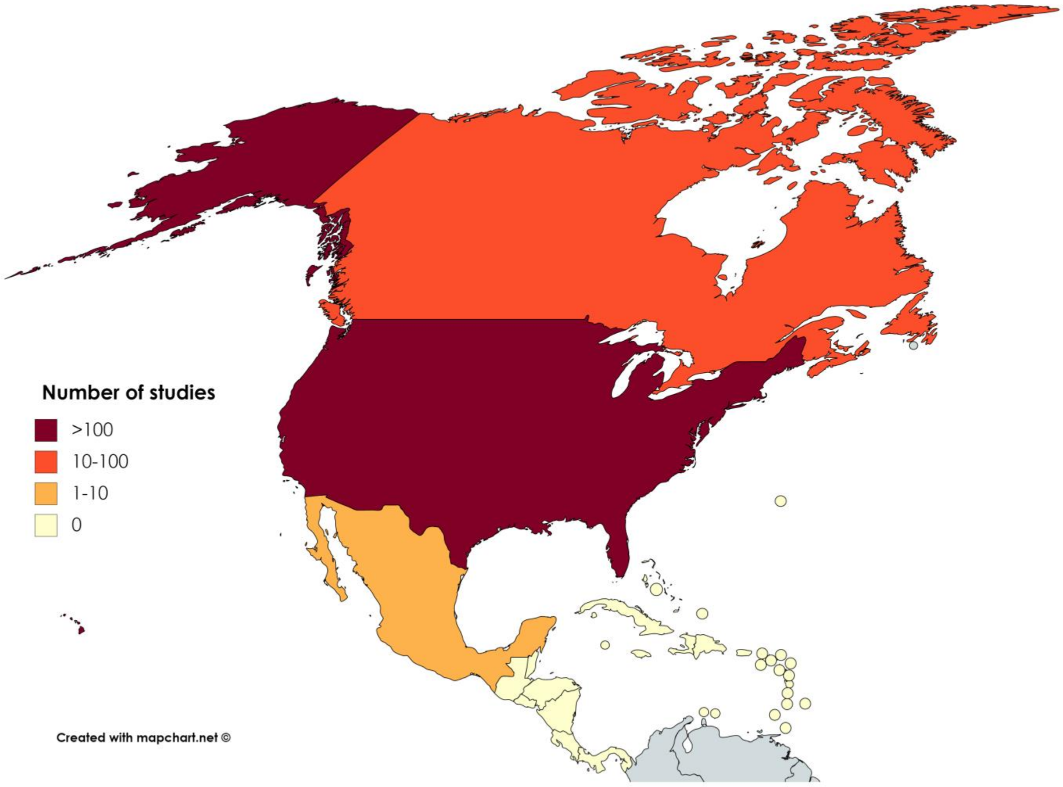

Figure A2.4 Laboratory RSV publications by country in North/Central America

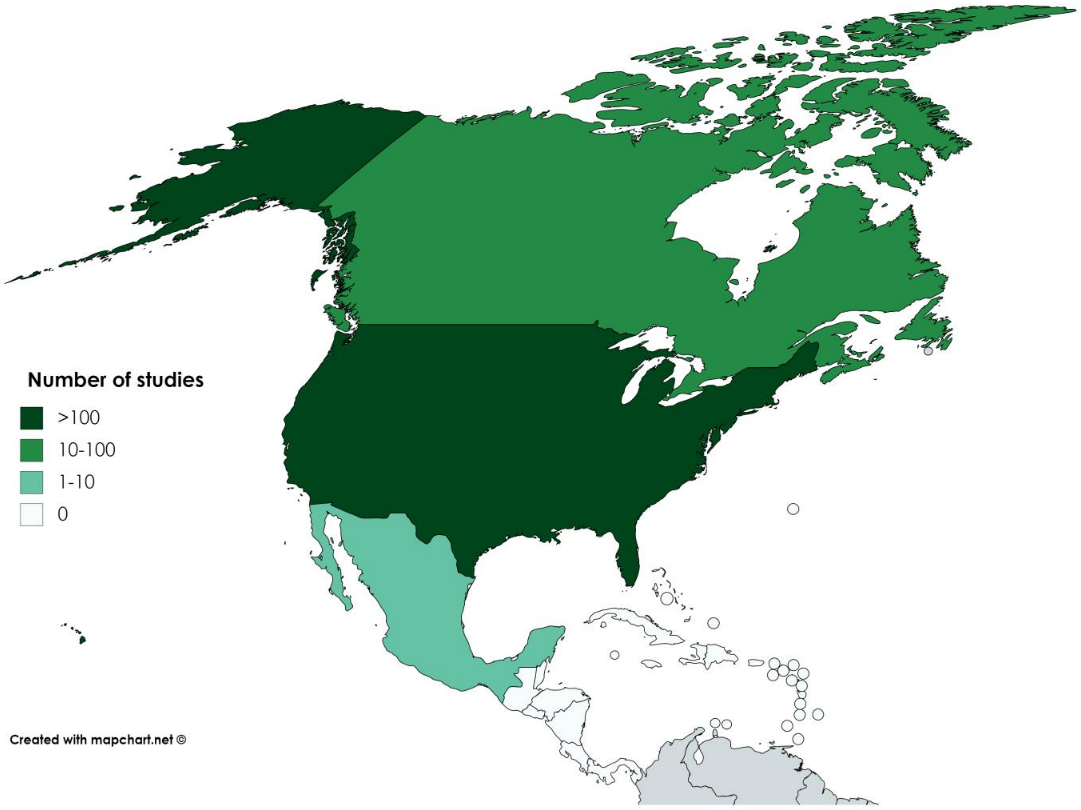

Number of studies
>100
10-100
1-10
0
Created with mapchart.net ©

Appendix 3 – Publications by US State

Table A3 Number of publications by US State

US State	Number of epidemiological publications	US State	Number of clinical publications	US State	Number of laboratory publications
**Maryland**	52	**New York**	19	**Maryland**	27
**Georgia**	37	**Maryland**	15	**Texas**	24
**New York**	12	**California**	14	**Georgia**	21
**Tennessee**	12	**Massachusetts**	11	**Tennessee**	19
**California**	11	**Ohio**	10	**Massachusetts**	18
**Massachusetts**	8	**Tennessee**	10	**Ohio**	17
**Florida**	6	**Texas**	10	**Michigan**	15
**Pennsylvania**	6	**Georgia**	8	**Iowa**	11
**Ohio**	5	**North Carolina**	7	**California**	8
**Texas**	5	**Missouri**	6	**Alabama**	7
**Colorado**	4	**Colorado**	4	**Pennsylvania**	6
**Michigan**	4	**New Jersey**	4	**New York**	5
**Washington**	4	**Washington**	4	**North Carolina**	5
**Alabama**	3	**Alabama**	3	**Virginia**	5
**Connecticut**	3	**Florida**	3	**Florida**	4
**Illinois**	3	**Pennsylvania**	3	**Colorado**	3
**Utah**	3	**Alaska**	2	**Washington**	3
**Wisconsin**	3	**Connecticut**	2	**West Virginia**	3
**Washington DC**	3	**Iowa**	2	**Kentucky**	2
**Louisiana**	2	**Kansas**	2	**Louisiana**	2
**Missouri**	2	**Minnesota**	2	**Missouri**	2
**Alaska**	1	**Washington DC**	2	**Oklahoma**	2
**Hawaii**	1	**Arizona**	1	**Wisconsin**	2
**Iowa**	1	**Arkansas**	1	**Washington DC**	2

US State	Number of epidemiological publications	US State	Number of clinical publications	US State	Number of laboratory publications
**Kentucky**	1	**Illinois**	1	**Illinois**	1
**New Hampshire**	1	**Louisiana**	1	**Indiana**	1
**New Jersey**	1	**Michigan**	1	**New Mexico**	1
**New Mexico**	1	**Oklahoma**	1	**Alaska**	0
**North Carolina**	1	**Virginia**	1	**Arizona**	0
**South Carolina**	1	**Wisconsin**	1	**Arkansas**	0
**Arizona**	0	**Delaware**	0	**Connecticut**	0
**Arkansas**	0	**Hawaii**	0	**Delaware**	0
**Delaware**	0	**Idaho**	0	**Hawaii**	0
**Idaho**	0	**Indiana**	0	**Idaho**	0
**Indiana**	0	**Kentucky**	0	**Kansas**	0
**Kansas**	0	**Maine**	0	**Maine**	0
**Maine**	0	**Mississippi**	0	**Minnesota**	0
**Minnesota**	0	**Montana**	0	**Mississippi**	0
**Mississippi**	0	**Nebraska**	0	**Montana**	0
**Montana**	0	**Nevada**	0	**Nebraska**	0
**Nebraska**	0	**New Hampshire**	0	**Nevada**	0
**Nevada**	0	**New Mexico**	0	**New Hampshire**	0
**North Dakota**	0	**North Dakota**	0	**New Jersey**	0
**Oklahoma**	0	**Oregon**	0	**North Dakota**	0
**Oregon**	0	**Rhode Island**	0	**Oregon**	0

US State	Number of epidemiological publications	US State	Number of clinical publications	US State	Number of laboratory publications
**Rhode Island**	0	**South Carolina**	0	**Rhode Island**	0
**South Dakota**	0	**South Dakota**	0	**South Carolina**	0
**Vermont**	0	**Utah**	0	**South Dakota**	0
**Virginia**	0	**Vermont**	0	**Utah**	0
**West Virginia**	0	**West Virginia**	0	**Vermont**	0
**Wyoming**	0	**Wyoming**	0	**Wyoming**	0

Footnote: Totals do not add up to the total number of clinical, epidemiological and laboratory studies reported for the USA elsewhere in this study due to publications where first and last authors were from different US states

Appendix 4 – Publications by institution

Table A4 Number of publications by institution

Research Institution	Epidemiological	Clinical	Laboratory	Overall
AstraZeneca/MedImmune	22	10	10	**42**
University Medical Center Utrecht	7	16	17	**40**
National Institutes of Health	14	0	21	**35**
Imperial College London	4	3	21	**28**
Emory University	8	3	12	**23**
Vanderbilt University	8	3	12	**23**
University of Michigan	4	2	15	**21**
Ohio State University	1	8	11	**20**
Centers for Disease Control & Prevention	10	3	4	**17**
Georgia State University	10	3	3	**16**
University of Texas Medical Branch Galveston	1	2	13	**16**
Children's Healthcare Atlanta	7	2	6	**15**
University of Toronto	11	1	3	**15**
University of Tennessee Health Science Center	4	5	6	**15**
Harvard Unviersity	6	6	2	**14**
Univeristy of Edinburgh	2	5	5	**12**
Radboud University Nijmegen Medical Center	0	7	5	**12**
John Hopkins University	9	3	0	**12**
Instituto de Salud Carlos III	1	0	10	**11**
University Groningen	7	3	1	**11**
McMaster University	8	3	0	**11**
University of Rochester	2	7	2	**11**
King's College London	2	8	0	**10**
Le Bonheur Children's Hospital	0	6	4	**10**
University of Washington	3	4	3	**10**
Karolinska Institutet	5	3	1	**9**
Rega Institute for Medical Research	0	7	2	**9**
US Food & Drug Administration	4	4	1	**9**
Erasmus Medical Center	4	3	1	**8**
Medical University of Graz	5	3	0	**8**
Boston University	0	1	7	**8**
Iowa State University	0	2	6	**8**
University of Saskatchewan	5	0	3	**8**
Institut Pasteur	0	0	8	**8**

Research Institution	Epidemiological	Clinical	Laboratory	Overall
National Institute for Public Health and the Environment (RIVM)	3	0	4	**7**
Turku University Hospital	3	4	0	**7**
University of Tampere	0	7	0	**7**
Baylor College of Medicine	1	3	3	**7**
University of Georgia	4	0	3	**7**
INRA, Unité de Virologie et Immunologie Moléculaires, Jouy-en-Josas	2	0	4	**6**

Footnote: Table displays only institutions who were identified as having 5 or more publications

Appendix 5 – Publications by funding body

Table A5 Number of publications by funding body

Funding Body	Epidemiological	Clinical	Laboratory	Total
National Institutes of Health	76	64	161	**301**
Astrazeneca/MedImmune	44	17	7	**68**
Abbott/Abbvie	31	25	1	**57**
European Commission	8	12	25	**45**
Medical Research Council	8	10	24	**42**
Wellcome Trust	6	5	21	**32**
Centers for Disease Control & Prevention	19	1	3	**23**
Canadian Institutes of Health Research	5	4	11	**20**
National Institute for Health Research (NIHR)	5	11	2	**18**
Bill & Melinda Gates Foundation	14	1	1	**16**
GlaxoSmithKline	6	7	3	**16**
Consejo Nacional de Ciencia y Tecnologia (CONACYT)	4	2	6	**12**
Emory University	5	2	5	**12**
Virgo Consortium	0	5	7	**12**
Georgia Research Alliance	7	1	3	**11**
Institute for the Promotion of Innovation by Science and Technology (IWT)	3	6	2	**11**
Novartis	6	4	1	**11**
University Medical Center Utrecht	0	9	2	**11**
March of Dimes	4	0	6	**10**
US Department of Veterans Affairs	1	1	8	**10**
Top Institute Pharma, the Netherlands	3	0	7	**10**
Sanofi	6	3	1	**10**
Vanderbilt University	3	1	5	**9**
Children's Healthcare of Atlanta	2	2	4	**8**
Flight Attendant Medical Research Institute	0	5	3	**8**

Funding Body	Epidemiological	Clinical	Laboratory	Total
Italian Ministry of Health	3	2	3	**8**
American Lebanese Syrian Associated Charities	4	2	1	**7**
Dutch Government	2	4	1	**7**
Sapienza Universita di Roma (Fondi Ricerche Universitari)	3	2	2	**7**
Agency For Healthcare Research and Quality (AHRQ)	5	1	0	**6**
American Heart Association	0	1	5	**6**
Asthma UK	0	4	2	**6**
Burroughs Wellcome Foundation	2	0	4	**6**
National Science Foundation	4	0	2	**6**
Netherlands Genomics Initiative (NGI)	0	2	4	**6**
US Department of Energy	3	1	2	**6**

Footnote: Table displays only funding bodies who were identified as providing funding for 5 or more publications

Appendix 6 – Highly cited RSV publications on Web of Science published between 2011-2015

Title	Author	Institution of first author	Institution of last author (if different)	Country	Year	Times cited (By 12 March 2018)
Respiratory Syncytial Virus and Recurrent Wheeze in Healthy Preterm Infants	Blanken MO	Univ Med Ctr Utrecht		Netherlands	2013	248
Structure-Based Design of a Fusion Glycoprotein Vaccine for RespiratorySyncytial Virus	McLellan JS	NIAID, Vaccine Res Ctr, NIH, Bethesda, MD		USA	2013	219
Hospitalizations Associated With Influenza and Respiratory Syncytial Virus in the United States, 1993-2008	Zhou H	Ctr Dis Control & Prevent, Influenza Div, Atlanta, GA		USA	2012	215
Progress in understanding and controlling respiratory syncytial virus: Still crazy after all these years	Colline PL	NIAID, Infect Dis Lab, NIH, Bethesda, MD 20892 USA	Inst Salud Carlos III, CIBER Enferrnedades Resp, Madrid 28220, Spain	USA, Spain	2011	214
Identification of nucleolin as a cellular receptor for human respiratorysyncytial virus	Tayyari F	Univ British Columbia		Canada	2011	153
Structure of Respiratory Syncytial Virus Fusion Glycoprotein in the Postfusion Conformation Reveals Preservation of Neutralizing Epitopes	McLellan JS	NIAID, Vaccine Res Ctr, NIH, Bethesda, MD		USA	2011	152
Cord Blood Vitamin D Deficiency Is Associated With Respiratory SyncytialVirus Bronchiolitis	Belderbos ME	Univ Med Ctr Utrecht		Netherlands	2011	149
Updated Guidance for Palivizumab Prophylaxis Among Infants and Young Children at Increased Risk of Hospitalization for Respiratory Syncytial VirusInfection	Brady MT	American Academy of Paediatrics		USA	2014	133

Respiratory Syncytial Virus-Associated Hospitalizations Among Children Less Than 24 Months of Age	Breese C	Univ Rochester, Sch Med & Dent, Dept Pediat, Rochester, NY	Ctr Dis Control & Prevent, Natl Ctr Immunizat & Resp Dis, Atlanta, GA USA	USA	2013	131
Biological challenges and technological opportunities for respiratory syncytial virus vaccine development	Graham BS	NIAID, Vaccine Res Ctr, NIH, Bethesda, MD 20892 USA		USA	2011	131
Respiratory Syncytial Virus-associated Hospitalizations Among Infants and Young Children in the United States, 1997-2006	Stockman LJ	Ctr Dis Control & Prevent, Div Viral Dis, Atlanta, GA 30333 USA		USA	2012	130
Structural basis for immunization with postfusion respiratory syncytial virusfusion F glycoprotein (RSV F) to elicit high neutralizing antibody titers	Swanson KA	Novartis Vaccines & Diagnost, Cambridge, MA 02139 USA		USA	2011	125
RNA Interference Therapy in Lung Transplant Patients Infected with Respiratory Syncytial Virus	Zamora MR	Univ Colorado, Denver Hlth Sci Ctr, Div Pulm Sci & Crit Care Med, Dept Med, Aurora, CO 80045 USA		USA	2011	122
Oral GS-5806 Activity in a Respiratory Syncytial Virus Challenge Study	DeVincenzo JP	Univ Tennessee, Sch Med, Memphis, TN USA	Gilead Sci Inc, Foster City, CA 94404 USA	USA	2014	118
TLR2/MyD88/NF-kappa B Pathway, Reactive Oxygen Species, Potassium Efflux Activates NLRP3/ASC	Segovia J	Univ Texas Hlth Sci Ctr San Antonio, Dept Microbiol &		USA	2012	101

Inflammasome<br>during Respiratory SyncytialVirus Infection	Immunol, San Antonio, TX 78229<br>USA.
